# Phenobarbital as alternate anticonvulsant for organophosphate‐induced benzodiazepine‐refractory status epilepticus and neuronal injury

**DOI:** 10.1002/epi4.12389

**Published:** 2020-04-14

**Authors:** Doodipala Samba Reddy, Dheepthi Perumal, Victoria Golub, Andy Habib, Ramkumar Kuruba, Xin Wu

**Affiliations:** ^1^ Department of Neuroscience and Experimental Therapeutics College of Medicine Texas A&M University Health Science Center Bryan TX USA

**Keywords:** DFP, nerve agents, organophosphate, phenobarbital, status epilepticus

## Abstract

**Objective:**

Organophosphates (OPs) such as diisopropylfluorophosphate (DFP) and soman are lethal chemical agents that can produce seizures, refractory status epilepticus (SE), and brain damage. There are few optimal treatments for late or refractory SE. Phenobarbital is a second‐line drug for SE, usually after lorazepam, diazepam, or midazolam have failed to stop SE. Practically, 40 minutes or less is often necessary for first responders to arrive and assist in a chemical incident. However, it remains unclear whether administration of phenobarbital 40 minutes after OP intoxication is still effective. Here, we investigated the efficacy of phenobarbital treatment at 40 minutes postexposure to OP intoxication.

**Methods:**

Acute refractory SE was induced in rats by DFP injection as per a standard paradigm. After 40 minutes, subjects were given phenobarbital intramuscularly (30‐100 mg/kg) and progression of seizure activity was monitored by video‐EEG recording. The extent of brain damage was assessed 3 days after DFP injections by neuropathology analysis of neurodegeneration and neuronal injury by unbiased stereology.

**Results:**

Phenobarbital produced a dose‐dependent seizure protection. A substantial decrease in SE was evident at 30 and 60 mg/kg, and a complete seizure termination was noted at 100 mg/kg within 40 minutes after treatment. Neuropathology findings showed significant neuroprotection in 100 mg/kg cohorts in brain regions associated with SE. Although higher doses resulted in greater protection against refractory SE and neuronal damage, they did not positively correlate with improved survival rate. Moreover, phenobarbital caused serious adverse effects including anesthetic or comatose state and even death.

**Significance:**

Phenobarbital appears as an alternate anticonvulsant for OP‐induced refractive SE in hospital settings. A careful risk‐benefit analysis is required because of negative outcomes on survival and cardio‐respiratory function. However, the need for sophisticated support and critical monitoring in hospital may preclude its use as medical countermeasure in mass casualty situations.

**Key Points**

- Phenobarbital produced a dose‐dependent reduction in OP‐induced refractory SE
- Higher doses of phenobarbital led to greater protection against SE and neuronal damage
- Higher doses of phenobarbital caused serious neurological morbidity or comatose state
- Phenobarbital appears as an alternate anticonvulsant for OP refractive SE but needs critical monitoring

## 1 INTRODUCTION

Organophosphate (OP) poisoning from pesticides and nerve agents is a lethal threat worldwide.^1^, ^2^, ^3^ OPs such as parathion and diisopropylfluorophosphate (DFP) are highly toxic chemicals that inhibit acetylcholinesterase (AChE) at central and peripheral cholinergic synapses. Thus, OP intoxication results in dramatic accumulation of acetylcholine leading to cholinergic crisis that manifests into predictable signs and symptoms, including constriction of the pupil, hypersecretion, bradycardia, tremors, fasciculations, persistent seizures, respiratory distress, and ultimately death.^4^, ^5^, ^6^, ^7^, ^8^ Among the most serious immediate results of OP poisoning is the onset of prolonged seizures known as status epilepticus (SE).^8^, ^9^ SE is a life‐threatening condition defined as recurrent or continuous self‐sustaining seizure activity lasting 5 minutes or longer in duration.^10^, ^11^ Among survivors, SE often result in marked neuronal injury and long‐term neurological dysfunction.^12^, ^13^ The current standard treatment protocol for acute OP intoxication includes administration of three drug types: atropine, pralidoxime (2‐PAM), and diazepam (or midazolam).^14^, ^15^ Atropine is an antagonist at muscarinic receptors that improves survival, and 2‐PAM reactivates AChE enzyme. However, both atropine and 2‐PAM have poor brain bioavailability and therefore offer only limited neurological protection.^16^

Benzodiazepines, such as diazepam and midazolam, are considered the first‐line treatment for SE.^16^, ^17^, ^18^ Although diazepam is the current standard anticonvulsant for OP seizures, there are many limitations associated with it.^19^, ^20^ First, its efficacy decreases substantially as time progresses after onset of OP‐induced SE; the treatment has been found to be ineffective at permanently terminating seizures after 40 minutes postexposure.^15^, ^21^ To effectively protect against seizures and SE, diazepam and midazolam must be administered within minutes of OP intoxication.^14^, ^22^, ^23^, ^24^, ^25^ In many medical emergencies, especially in mass casualty scenarios, this timeline is not feasible. Second, resistance to treatment with benzodiazepines is a serious concern,^23^ as confirmed for diazepam^24^ and midazolam.^25^ These reports reinforce the need for developing better anticonvulsants. Phenobarbital is a second‐line treatment for SE, usually after lorazepam, diazepam, phenytoin, or midazolam have failed to stop SE within 30 minutes after a patient is admitted to emergency room.^14^, ^16^, ^17^, ^26^, ^27^ Phenobarbital has a long history, first used as broad‐spectrum antiepileptic and then as anticonvulsant drug given in refractory SE.^26^, ^27^ Phenobarbital causes side effects ranging from renal failure, myocardial impairment, and sedation, to respiratory depression depending on the dose, including depression in the level of consciousness, hypotension, paralysis, and coma state at high doses or from drug accumulation due to its long half‐live of 3‐7 days.^14^, ^26^, ^28^, ^29^ Patients administered therapeutic or high dosages of phenobarbital for OP‐induced SE must often remain under clinical observation for airway and cardio‐respiratory vital functions, including continuous monitoring of blood pressure and respiratory rate. Because of the strong anticonvulsant property of phenobarbital, it could be used as an alternative to diazepam or midazolam to control SE after nerve agent exposure.^4^, ^30^, ^31^, ^32^ However, the risk vs benefit of phenobarbital therapy, especially neurological morbidity of varying phenobarbital dosage, for OP‐induced SE remains unclear.

In this study, we characterized the dose‐dependent efficacy of phenobarbital treatment at 40 minutes post‐DFP intoxication. Comparative seizure protection was determined by video‐EEG techniques, neuronal protection was assessed by neuropathology quantifications of injured neurons, and neurodegeneration through histology and unbiased stereology.

## 2 MATERIALS AND METHODS

### 2.1 Animals

Adult male Sprague‐Dawley rats (3 months old; 250‐300 g) (Taconic Farms) were used in the study. All procedures were performed in compliance with the guidelines of NIH Guide for the Care and Use of Laboratory Animals under a protocol approved by the university's Institutional Animal Care and Use Committee.

### 2.2 EEG electrode implantation

Rats were anesthetized with a mixture of ketamine (100 mg/kg) and xylazine (10 mg/kg) administered via intraperitoneal injection. Two metal EEG recording electrodes with mounting screws (Plastics One, Roanoke, VA) were placed epidurally using a stereotaxic apparatus: one over the right frontoparietal cortex and one over the left cerebellum (reference electrode). Next, a bipolar electrode (PlasticsOne) was placed into the right dentate gyrus in the following areas: anteroposterior (4 mm posterior to bregma), mediolateral (2.3 mm lateral to midline), and dorsoventral (3.4 mm deep). Ten days after the surgery, experiments began.

### 2.3 DFP exposure and video‐EEG recording

**Figure 1:**
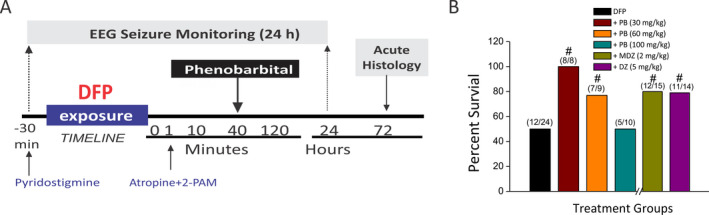
Diisopropylfluorophosphate (DFP) model of OP intoxication in rats. A, DFP‐induced SE model experimental paradigm. Rats were pretreated with pyridostigmine bromide (0.026 mg/kg, im) 30 min before DFP (3.2 mg/kg, s.c.) exposure. One minute following DFP injection, rats were injected with pralidoxime (2‐PAM, 25 mg/kg, im) and atropine methyl nitrate (2 mg/kg, im) to increase survival rates. Phenobarbital (30‐100 mg/kg, im) was given at 40 min post‐DFP exposure. All cohorts were monitored continuously up to 24 h post‐DFP by recording behavioral and EEG seizure activity. Brains were collected for histology at 72 h post‐DFP exposure. B, Comparative 24 h's survival outcomes in various subgroups of phenobarbital (PB)‐, midazolam (MDZ)‐, and diazepam (DZ)‐treated animals. In the DFP‐alone group, survival rate was 50% in this experimental cohort (n = 24; 12 out of 24 rats survived). Survival outcome was calculated 24 h after exposure to DFP by the nonparametric Wilcoxon signed‐rank test. #*P* < .05 vs DFP group (n = 8‐24 rats/group; exact group sizes are listed in B)

The overall protocol for DFP and drug treatment is illustrated in Figure 1A. We used the DFP method as described previously,^25^ which is comparable to the protocol that was adapted by many NIH CounterACT investigators.^33^, ^34^ Rats were pretreated with an intramuscular (im) injection of pyridostigmine bromide (0.026 mg/kg) 30 minutes prior the subcutaneous DFP (3.2 mg/kg, s.c.) exposure. DFP is a very potent neurotoxin with LD_50_ of 6 mg/kg (oral) in rats. Approximately one minute after exposure, rats received atropine methyl nitrate (2 mg/kg, im; half‐life, 0.5 to 4 hours) and pralidoxime chloride (25 mg/kg, im; half‐life, 1.4 hours). Animals were observed for seizures and SE for 24 hours postexposure and euthanized at 72 hours postexposure (Figure 1A). DFP‐induced SE was defined as the appearance of large amplitude, repetitive discharges (>0.5 Hz with at least double the amplitude of the background activity). The behavioral seizures were monitored and classified according to the 0‐5 Racine scale of epileptic seizure stages.^35^ An additional cohort of vehicle‐treated rats, with and without electrode implant, served as controls for the test drug.

### 2.4 Phenobarbital treatment

There was one DFP‐only group (n = 24) that received a vehicle injection in a volume equal to phenobarbital. There were multiple phenobarbital (30, 60 and 100 mg/kg, im) treatment groups that received a single drug injection (phenobarbital 30 mg/kg, n = 8; phenobarbital 60 mg/kg, n = 9; phenobarbital 100 mg/kg, n = 10) at 40 minutes post‐DFP exposure. For comparison and confirmation of benzodiazepine resistance of SE in the OP intoxication model, a separate subgroup was treated with the benzodiazepine diazepam (5 mg/kg, im)^24^ or midazolam (2 mg/kg, im)^25^ and compared the results with phenobarbital (60 mg/kg) at 40 minutes after DFP exposure.

### 2.5 Brain perfusion and tissue processing

Rats were deeply anesthetized at 72 hours postexposure as described and then transcardially perfused with normal saline followed by 4% paraformaldehyde solution in sodium phosphate buffer (pH 7.4). The brain were excised and postfixed as described previously.^24^, ^25^

### 2.6 Analyses of the extent of neurodegeneration

To analyze the overall neurodegeneration, DFP‐exposed control group and DFP‐exposed groups treated with phenobarbital (n = 5‐6) were euthanized at 3 days post‐treatment and the brain tissues processed for cresyl violet (Nissl) staining and Fluoro‐Jade B (FJB(+)) immunostaining as described previously.^25^ The extent of neurodegeneration was assessed as per the severity of cells loss on a scale of 0 (no neuropathology) to 4 (severe neuropathology). Briefly, the tracings from the control Nissl‐stained sections were overlapped on the Nissl or FJB‐stained sections. Neuropathology scores were based on the severity of Nissl (cell loss) and FJB(+)‐stained neurons to the cells in the control group of Nissl staining: 0 = no neuropathology (0% staining); 1 = minimal neuropathology (1%‐10% staining); 2 = mild neuropathology (11%‐25% staining); 3 = moderate neuropathology (26%‐45% staining); and 4 = severe neuropathology (>45% staining). Such assessment has been previously shown to produce results that are in concurrence with quantitative stereology counting^25^, ^36^. For both FJB(+) and Nissl immunohistochemistry, at least 3 sections from each of the regions of interest (ROI) were reviewed from 9 slices per animal, totaling 27 sections per ROI per animal. These sections were averaged together to create each rat's score per ROI. Regions of interest include the following: thalamus, hypothalamus, entorhinal cortex, somatosensory cortex, piriform cortex, and amygdala. These sections were reviewed and finally imaged for publication using a 10× objective. Rats that were not exposed to DFP, but instead received a vehicle injection in a volume equivalent to the drug exposure groups, served as baseline controls.

### 2.7 Stereology quantification

Design‐based stereology was used to quantify the total number of neurons and percentage of neuroprotection in various immunohistochemistry sections, as previously described.^37^ The absolute cell counts in the hippocampus were calculated using the optical fractionator component of the Visiopharm software.^25^ The neuron numbers were quantified at 10% of total region area for FJB(+) cells at 60x objective lens in the CA1, CA3, and dentate gyrus (DG) subfields. The total area was increased to 15% for FJB(+) counts in the CA2 and DH subfields. The total area selection was based on the relative density cells in these regions to ensure optimal sampling for stereological cell counts. For accurate volume estimation, more than 200 points covering 100% tissue sections at 10x objective lens were counted in each region of interest.^25^, ^37^, ^38^, ^39^

### 2.8 Experimental outcomes and analysis

The study was designed to evaluate the efficacy of phenobarbital as an anticonvulsant antidote for OP intoxication (Figure 1A). Rats were randomly assigned to groups using the randomization sequence generation. The sample size (n) needed for each experiment was calculated using the power analysis for obtaining statistically significant (α = 0.05) outcomes based on Lamorte's power calculations, the magnitude of effect observed and its variability in our preliminary studies, and published reports.^25^, ^40^ The power and sample size were computed based on the proposed statistical tests including one‐way and two‐way repeated measures ANOVA for neuropathological results. A sample size of 8 (n = 8) was found to be adequate for the dose‐response study of phenobarbital in DFP seizure model. A sample size of five or more was found to be sufficient for neuroprotection outcomes. Test drugs were evaluated in a dose‐dependent fashion at 40 minutes after DFP exposure, which is considered a critical period and simulation of practical therapeutic window for first responders for emergency care in the case of chemical incidents. Behavioral and EEG seizures were recorded continuously for 24 hours. Acute histological outcome was assessed at 72 hours after DFP exposure. Appropriate controls were utilized, including (a) vehicle‐treated (DFP‐exposed) rats as control for test drug, and (b) non‐DFP rats as additional baseline control group. We analyzed two outcomes of phenobarbital treatment effectiveness: (a) anticonvulsant efficacy and (b) neuroprotectant efficacy.

### 2.9 Test drugs

Phenobarbital injection (100 mg/mL) was purchased from Paterson Veterinary. DFP, atropine, and 2‐PAM were purchased from Sigma‐Aldrich. Ketamine, xylazine, heparin, and dextrose sterile injection solutions were procured from Paterson Veterinary. Phenobarbital was diluted in sterile saline. DFP was diluted in cold sterile PBS solution.

### 2.10 Statistical analysis

The statistical values are expressed as the mean ± SEM. In all statistical tests, statistically significant differences were set at *P* < .05. Statistical comparisons of seizure activity and neuroprotection outcomes were performed with one‐way or two‐way repeated measures analysis of variance (ANOVA) as appropriate. Post hoc analyses were carried out to identify specific differences using Tukey's honestly significant difference (HSD) for multiple comparisons. Nonparametric outcomes, such as mortality/survival outcomes, were compared between groups using the Wilcoxon signed‐rank test, as outlined previously.^41^, ^42^ Behavior seizure score and neuropathology score outcomes were analyzed with the nonparametric Kruskal‐Wallis test followed by the Mann‐Whitney U test. Statistical tests were performed using SAS software (SAS Institute Inc) and Macrocal Origin 8 (OriginLab Corporation).^25^ Group sizes were dependent on survivability following DFP exposure. For all EEG analysis and seizure behavior, group size ranged between n = 5‐12 (DFP, n = 12; phenobarbital 30 mg/kg, n = 8; phenobarbital 60 mg/kg, n = 7; phenobarbital 100 mg/kg, n = 5; midazolam, n = 12; diazepam, n = 11). These exact group sizes are reiterated in Figure 1B. Throughout the remainder of this paper, we have simplified group size as n = 5‐12. For all immunohistochemistry analysis, group size ranged between n = 5‐6 (DFP, n = 6; phenobarbital 30 mg/kg, n = 5; phenobarbital 60 mg/kg, n = 6; phenobarbital 100 mg/kg, n = 5; midazolam, n = 5; diazepam, n = 5). Additionally, a naïve control group was used to compare the extent of damage after DFP exposure (n = 8). These group sizes were found to be sufficient to obtain statistically significant results according to Lamorte's power calculations, as described above.

## 3 RESULTS

### 3.1 Effect of phenobarbital on DFP‐induced acute seizures and SE

To determine the efficacy of phenobarbital, we tested it at 30, 60, and 100 mg/kg doses given at 40 minutes after DFP intoxication (Figure 1A). Exposure of rats to DFP triggered rapid behavioral cholinergic hyperactivation within 2‐3 minutes. It was characterized by excessive salivation, twitches and behavioral seizures with stage 1 or 2, such as bouts of chewing activity and intermittent head tremors. This activity progressed into explosive tonic‐clonic motor convulsions and finally into SE (stage 5) at 8 to 10 minutes post‐DFP. The 24‐h survival rate was 50% after DFP (n = 12/24) without any protective treatment that is consistent with the survival rate in DFP models as described in prior studies^24^, ^25^. The survival rates for phenobarbital‐treated groups are listed in Figure 1B. Higher survival rates were evident in rats treated with phenobarbital 30 mg/kg (100% survival, n = 8 survived out of 8) and 60 mg/kg (77% survival, n = 7/9) after DFP exposure (*P* < .05 vs DFP‐alone group). However, at 100 mg/kg phenobarbital dosage, the survival rate (50%, n = 5/10) was similar to DFP group (50%, n = 12/24; Figure 1B). The comparative survival rate of benzodiazepine‐treated groups, such as diazepam (79% survival, n = 11/14) and midazolam (80% survival, n = 12/15), is also provided in Figure 1B.^24^, ^25^

**Figure 2:**
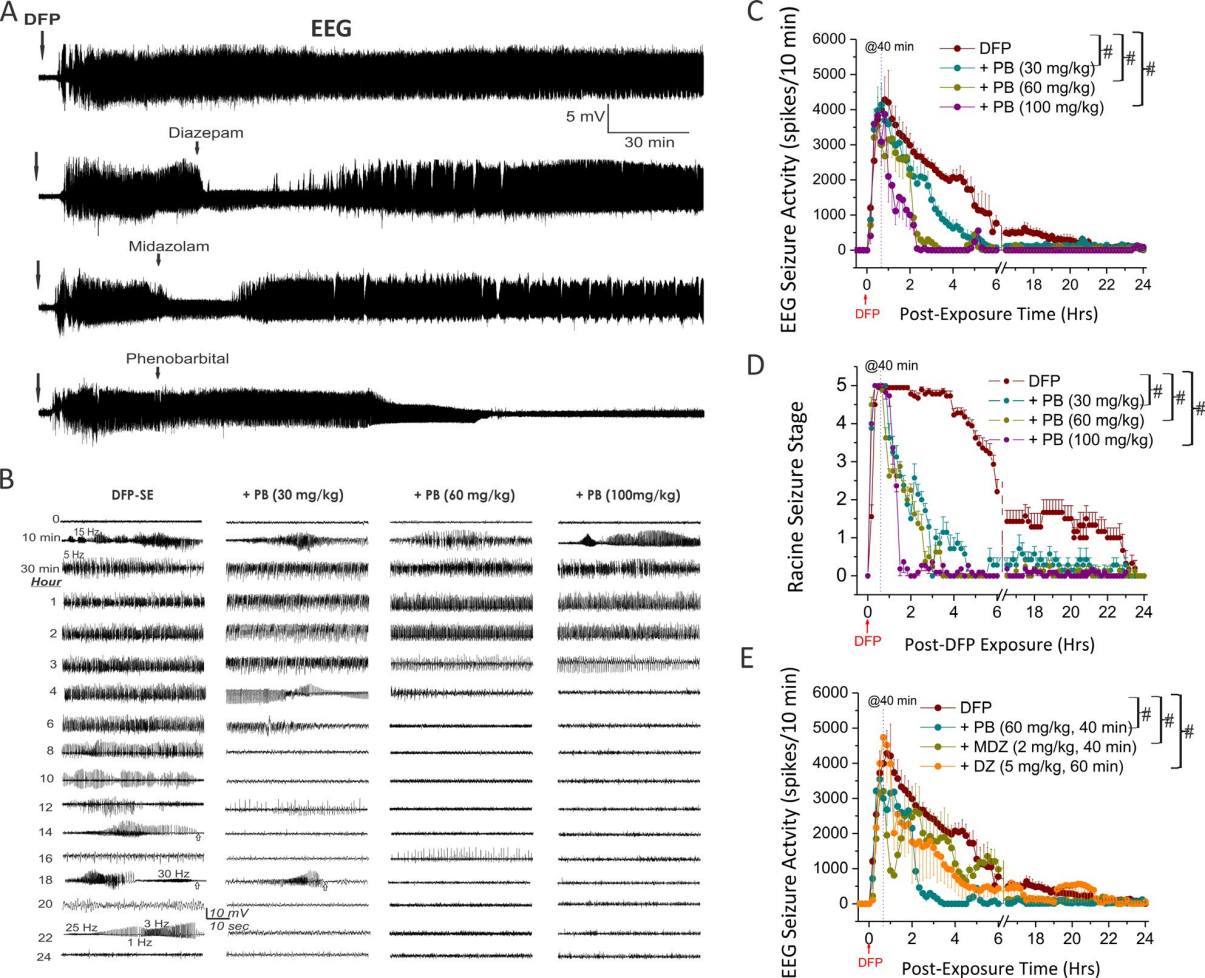
Effect of phenobarbital (PB) on DFP‐induced electrographic seizures and status epilepticus in hippocampus in rats. A, Comparative effect of benzodiazepines and phenobarbital on DFP‐induced status epilepticus (SE). EEG traces depicting electrographic seizure activity over 4‐h period from a depth electrode in the hippocampus in rats following DFP. PB at 100 mg/kg terminated SE, while midazolam (2 mg/kg, im, 40 min post‐DFP) and diazepam (5 mg/kg, im, 60 min post‐DFP) initially suppressed SE but rebounded with strong SE activity. B, EEG traces represent 1 min epochs. Typical seizures began ~ 5‐10 min post‐DFP and include the following: the initial spikes increase in amplitude with time and dramatic drop off following the seizure, generally back to baseline with a significant decline in the amplitude of the signal following the completion of a EEG seizure activity (depicted by arrows in traces 14‐ and 18‐h epochs); the frequency varies from 0.5 to 30 Hz. Chart depicts epochs of EEG traces over 24‐h period for DFP group (n = 12) and three dosages of PB. DFP group exhibited SE for over 12 h, while the 60 and 100 mg/kg PB groups offered significant seizure protection within 3‐4 h postexposure. Scale bar is 10 mV and 10 sec. C, Graph depicting mean EEG seizure activity over 24‐h periods following initial exposure to DFP. PB at 100 mg/kg (n = 4) significantly decreases seizure activity after 1 h; 60 mg/kg dosage (n = 5) does so after 2 h, and 30 mg/kg (n = 6) after 3 h. PB at 60 and 100 mg/kg groups offered significant seizure protection. Repeated measures analysis of variance (ANOVA) and post hoc analyses were carried out to identify specific differences using Tukey's honestly significant difference (HSD) for multiple comparisons, as appropriate. D, Time course of behavioral seizure suppression by PB treatment. Behavior seizures were significantly inhibited by PB application (Kruskal‐Wallis test followed by the Mann‐Whitney U test). Rats were monitored for 24 h after exposure to DFP. E, Graph depicting comparison effects of PB, midazolam (MDZ, n = 7), and diazepam (DZ, n = 6) 40‐60 min post‐DFP on EEG seizure activity over 24‐h periods. PB at 60 mg/kg significantly decreases seizure activity than MDZ and DZ during the periods of 2‐4 h post‐DFP challenge. Repeated measures analysis of variance (ANOVA) and post hoc analyses were carried out to identify specific differences using Tukey's honestly significant difference for multiple comparisons, as appropriate. Each bar represents the mean ± SEM. #*P* < .05 vs DFP alone (or *P* < .05 between phenobarbital at 60 mg/kg vs midazolam or diazepam groups in E).

EEG recordings from the hippocampus showed the progression of DFP‐induced persistent spiking indicating SE activity (Figure 2A). The spikes began 5‐10 minutes post‐DFP with a frequency of 0.5 to 30 Hz (Figure 2B). The SE was very intense and persistent for over 3 hours after DFP (Figure 2A,B). The EEG recordings from the cortex showed the progression of DFP‐induced SE‐like spiking activity similar to recording from hippocampus (data not shown). EEG alterations were correlated with behavioral seizure manifestations, such as unilateral or bilateral forelimb clonus and rearing and falling, which were rated by the Racine scale (Figure 2D). Seizures and SE were attenuated after phenobarbital (30‐100 mg/kg) administered at 40 minutes post‐DFP. Administration of phenobarbital, at dose that is considered human equivalent dose, at 40 minutes post‐DFP resulted in a slower onset of termination of behavioral and electrographic SE (Figure 2A,E). The correlation between behavioral seizure score and EEG activity was statistically significant (*P* < .01) during a 4‐hour recording after DFP exposure in both DFP control and phenobarbital groups (Figure 2C,D; n = 5‐12 rats/group). However, there was a rebound effect as seizures returned in 30 mg/kg phenobarbital group within 1‐2 hours (Figure 2B,C). When 30 mg/kg phenobarbital was administered 40 minutes post‐DFP, both behavioral and electrographic SE were only temporary decreased or resulted in marginal reduction in SE activity, indicating that phenobarbital at 30 mg/kg has limited effect at preventing seizures, but it significantly increased survival rate after exposure to DFP (*P* < .05 vs DFP alone; Figure 1B).

Diazepam, midazolam, and phenobarbital are clinically used for the management of SE ^14^, ^17^, ^21^, ^43^. We compared these three drugs on DFP‐induced SE at 40 minutes post‐DFP (Figure 2A). We utilized the electrophysiological outcomes as illustrated in the EEG traces depicting electrographic seizure activity over 4‐h period following DFP. Phenobarbital (100 mg/kg) at slow onset, terminated SE, while midazolam (2 mg/kg, n = 7) and diazepam (5 mg/kg, n = 6) at quick onset, initially suppressed SE but rebounded with strong SE activity 30 to 60 minutes later (Figure 2A,E). The 24‐h survival rate for midazolam and diazepam was about 80% at 40‐60 minutes post‐DFP (Figure 1B). In contrast to benzodiazepines, phenobarbital produced robust and prolonged protection against SE, and significantly (*P* < .05) decreased seizure activity when SE showing resistance to the diazepam and midazolam during the periods of 2‐6 hours post‐DFP exposure (Figure 2A,E). Overall, phenobarbital treatment terminated seizures more effectively than benzodiazepines at 60 and 100 mg/kg dose but with negative impact on neurological function and survival rate.

All groups were observed for neurological and clinical signs of adverse effects following phenobarbital treatment. All treated animals in the high‐dose group (100 mg/kg) showed strong hypoactivity and sedation starting at 30 minutes postdose, and intense ataxia and reduced respiration at 2 hours postdose. These effects were drug therapy‐related and pharmacologically anticipated clinical effects of phenobarbital. Some of these animals were normal by 6 hours postdose on day 1 and until the end of the 24‐h observation period. A few animals deceased overnight most likely due to respiratory distress (Figure 1B), indicating the risk from higher dose of animal morbidity or mortality. The low‐dose group (30 and 60 mg/kg) animals exhibited moderate signs with a better survival rate (Figure 1B).

### 3.2 Effect of phenobarbital on DFP‐induced acute FJB(+) neuronal injury and necrosis in the hippocampus

**Figure 3:**
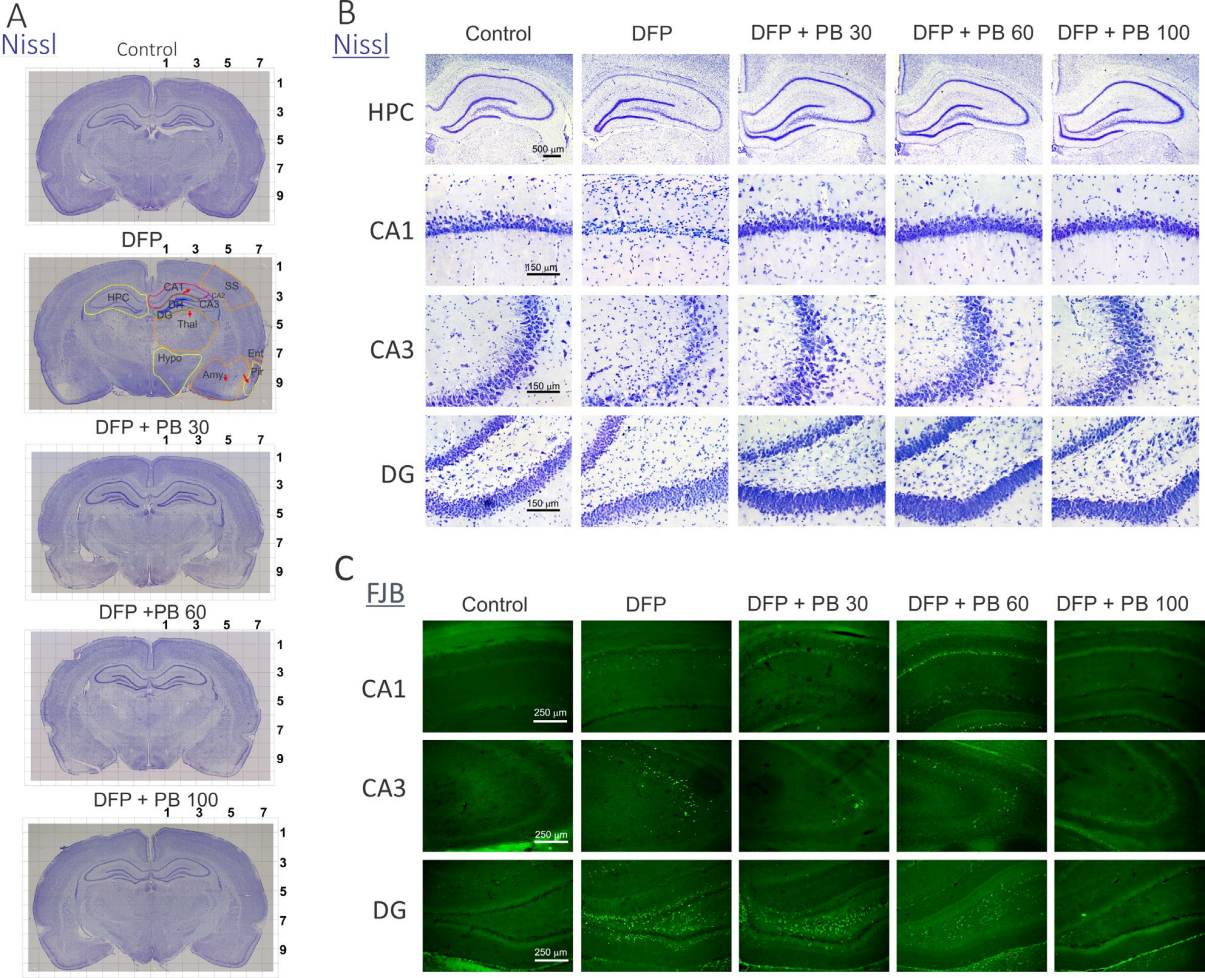
Dose‐dependent protective profile of phenobarbital (PB; 30, 60, and 100 mg/kg) on DFP‐induced acute neuronal injury in the hippocampus subfields. A, Nissl‐stained images of coronal brain slices. Notable degeneration of neurons can be seen in within both hemispheres of the DFP group (red arrows indicating representative degenerative areas one one side). The digit numbers in the top and right side indicate the distance to/from the midline (mm). HPC: hippocampus; Thal: thalamus; Amy: amygdala; Pir: piriform cortex; Hypo: hypothalamus, amygdala (Amy); SS: somatosensory cortex; Ent: entorhinal cortex. Light gray grid unit = mm. B, Nissl‐stained images of hippocampus (HPC, objective 1.25×) and regions of CA1, CA3 and dentate gyrus (DG) (objective 20×). There is greater neurodegeneration within the DFP group when compared to all treated cohorts. C, Representative FJB confocal images of degenerative neurons in hippocampal regions for control, DFP, and therapeutic phenobarbital dosages 40 min post‐DFP (objective 10×). There are few degenerative neurons in phenobarbital groups

**Figure 4:**
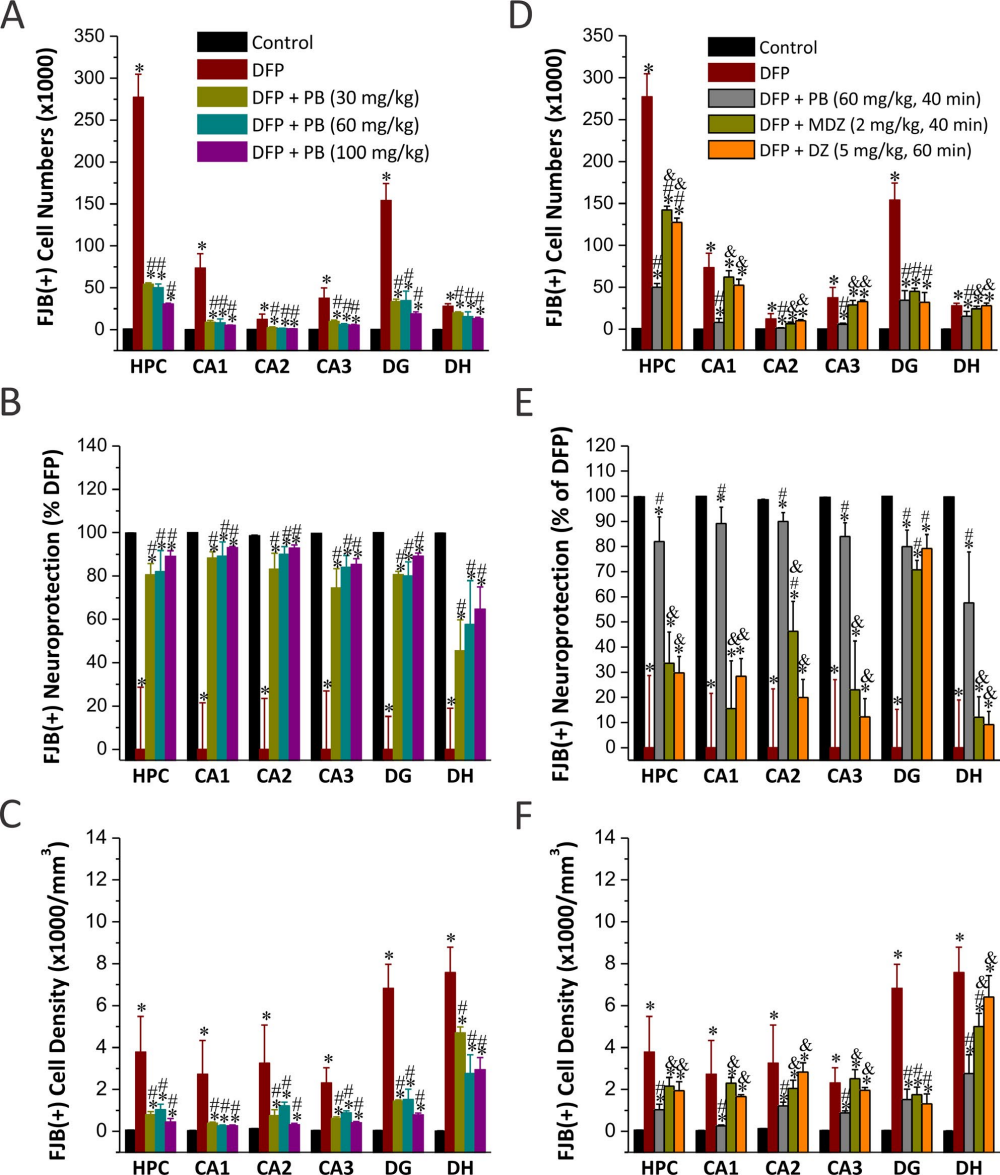
Stereological quantification of phenobarbital (PB) on DFP‐induced acute neuronal injury with FJB(+) staining in the hippocampus subfields. A, Stereology data showed significant increased FJB (+) cell numbers in DFP groups (n = 6) in CA1, CA2, CA3, DG, and dentate hilus (DH). B, Normalized percent neuroprotection by PB with reference to FJB(+) cells in DFP group. Normalized neuroprotection was calculated using the untreated DFP‐exposed group as the baseline (0% protection). In this estimate, control group not exposed to DFP was rated as 100% protected due to the lack of any FJB(+) cells in any regions. C, FJB(+) cell density by stereology in hippocampal subfields. Comparative stereology data showed significant decrease in FJB (+) cell numbers (D), increased normalized percent neuroprotection (E), and decreased cell density (F) in DG region from PB (60 mg/kg, n = 6), midazolam (MDZ, n = 5), and diazepam (DZ, n = 5) at 40‐60 min post‐DFP. However, there was significantly reduced neuroprotection in other hippocampal regions examined in MDZ and DZ groups. Normalized neuroprotection was calculated same as in B. Value bars represent the mean ± SEM. *P < .05 vs control (n = 12); #*P* < .05 vs DFP group (n = 8); ^&^ *P* < .05 vs PB 60 mg/kg group (n = 5; two‐way repeated measures ANOVA and post hoc Tukey's honestly significant difference test)

DFP intoxication causes massive neuronal injury (~41% of control, control group = 12) and remarkable neurodegeneration in many brain regions including hippocampus subfields (Figure 3A,B)^25^. To determine the extent of DFP‐induced neurodegeneration in the hippocampus of animals receiving vehicle or phenobarbital, FJB (+) staining was used to assess the extent of neuronal injury at 72 hours after DFP exposure (Figure 3C). In FJB‐stained sections, the degenerating neurons in the brain sections exhibited a bright green fluorescence in the hippocampus subfields CA1, CA3, and DG subregions (Figure 3C). Since FJB predominantly stains cell body or necrotic cells, we selected to illustrate the damaged neurons at low magnification and stereological quantification of damaged cell counts. Neuropathological analysis was performed by unbiased stereology in the groups that received 30 (n = 5), 60 (n = 6), and 100 mg/kg (n = 5) phenobarbital after DFP exposure. Normalized neuronal protection was determined based on comparisons with the untreated DFP‐exposed group. All groups were also compared to naïve control animals (n = 8). Stereological counts for the number of FJB(+) cells showed that DFP exposure resulted in massive and significant increase (*P* < .0001) in the number of FJB(+) cells in the whole hippocampus and its subfields (Figure 4). Phenobarbital at all doses showed a marked decrease in neuronal damage in comparison with the DFP cohort as evident from the ANOVA comparison (*P* < .01 vs DFP alone; Figure 4A). Phenobarbital treatment produced a dose‐dependent neuroprotection when administered post‐DFP with significantly attenuation of DFP‐induced neuronal degeneration (60%‐90% protection) at 60 mg/kg and 100 mg/kg cohorts in many hippocampus subfields (Figure 4B), including CA1 (*P* < .001), CA2 (*P* < .001), CA3 (*P* < .001), dentate gyrus (*P* < .001), and dentate hilus (*P* < .05) regions. The extent of neuronal injury in animals treated with 60 and 100 mg/kg phenobarbital after DFP groups was relatively less compared to low dose of 30 mg/kg phenobarbital, as evident in the absolute FJB(+) cell numbers (Figure 4A), percent protection (Figure 4B), and cell density (Figure 4C). Normalized neuronal protection in the groups that received phenobarbital after DFP exposure was determined based on comparisons with the untreated DFP‐exposed group (as 0% percentage), while control group without FJB(+) staining was considered as 100% protection. The volumes for hippocampus (74.1 ± 0.6 mm^3^) and its subfields of CA1 (27.1 ± 1.5 mm^3^), CA2 (3.8 ± 0.4 mm^3^), CA3 (17.7 ± 0.2 mm^3^), DG (25.5 ± 0.6 mm^3^), and DH (4.9 ± 0.1 mm^3^) in control (n = 12) had no significant difference (*P* = .1) among all examined groups, indicating similar tissue thickness for stereology counts and there was no change in tissue volumes after DFP exposure (data not shown). This neuroprotective effect by phenobarbital (60 mg/kg) is far superior and statistically significant (*P* < .05) in most brain regions as compared to diazepam (5 mg/kg, n = 5) and midazolam (2 mg/kg, n = 5) cohorts, as evident from the FJB(+) cell numbers (Figure 4D), percent protection (Figure 4E), and cell density (Figure 4F). Thus, these results suggest higher dosages of phenobarbital provided significant neuronal protection from DFP‐induced necrosis and neurotoxicity. However, higher dosages of phenobarbital did not positively correlate with improved survival rates (Figure 1B). The decrease in survival rates possibly correlated with serious adverse effects from higher dosages of phenobarbital including respiratory depression, anesthetic or comatose state, and even death.

### 3.3 Effect of phenobarbital on DFP‐induced acute FJB (+) neuronal injury and necrosis in the extrahippocampal regions

**Figure 5:**
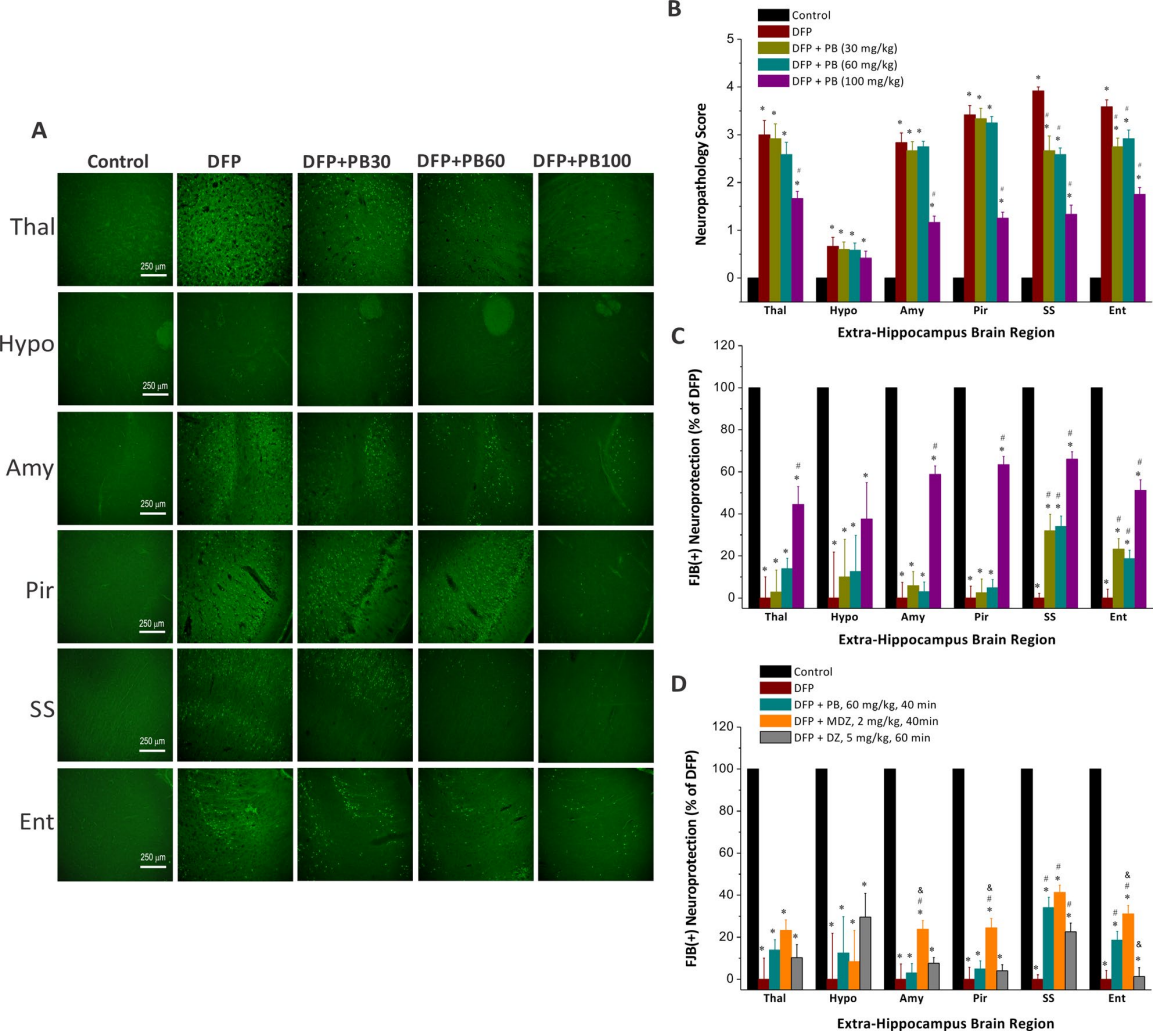
Dose‐dependent protective profile of phenobarbital (PB) on DFP‐induced acute neuronal injury in the extrahippocampal regions. A, Representative FJB confocal images of degenerative neurons in extrahippocampal for control (n = 12), DFP (n = 6), and therapeutic phenobarbital dosages 40 min post‐DFP. FJB(+) staining showed moderate‐to‐severe neurodegeneration in DFP group. Few degenerative neurons were found in 100 mg/kg phenobarbital group in the extrahippocampal regions including thalamus (Thal), hypothalamus (Hypo), amygdala (Amy), piriform cortex (Pir) somatosensory cortex (SS), and entorhinal cortex (Ent) regions. B, The bar chart depicts neuropathology scores in these regions and represents severity level of FJB(+) staining neurons. DFP exposures were associated with severe damage with high neuropathology score. C, Normalized percent neuroprotection in extrahippocampal brain regions from rats treated with PB dosages 40 min post‐DFP. Normalized neuroprotection was calculated using the untreated DFP‐exposed group as the baseline (0% protection). D, Comparative normalized percent neuroprotection in extrahippocampal brain regions from rats treated with PB, midazolam (MDZ), and diazepam (DZ) at 40‐60 min post‐DFP. Value bars represent the mean ± SEM. *P < .05 vs control (n = 12); #*P* < .05 vs DFP group (n = 8); ^&^ *P* < .05 vs PB 60 mg/kg group (n = 5; Kruskal‐Wallis test followed by the Mann‐Whitney U test)

The neuronal injury induced by DFP was analyzed in other extrahippocampal brain regions including the thalamus, hypothalamus, amygdala, piriform cortex, somatosensory cortex, and entorhinal cortex (Figure 5A). A simple neuropathology‐based relative quantification of neuronal injury was used to assess neuronal injury in the extrahippocampal regions, as described previously.^25^ In the DFP cohort (n = 6), significant neuronal injury and necrosis were seen in the thalamus, piriform cortex, amygdala, somatosensory cortex, and entorhinal cortex (Figure 5A). The hypothalamus had the lowest FJB (+) cells compared to the other brain regions (Figure 5A,B). The extent of neuronal injury was significantly lower (*P* < .05) in animals treated with high‐dose (100 mg/kg, n = 5) phenobarbital as compared to other doses (Figure 5). However, there was limited or no significant (*P* = .1) protection in low (30 mg/kg, n = 5)‐ and medium (60 mg/kg, n = 6)‐dose treatment groups in most extrahippocampal regions (Figure 5B,C). Normalized neuronal protection in the groups that received phenobarbital after DFP exposure was determined based on comparisons with the untreated DFP‐exposed group, while the control group was as 100% protection. Administration with benzodiazepines, diazepam (n = 5) or midazolam (n = 5) resulted in similar neuroprotection in most extrahippocampal regions to that of phenobarbital (60 mg/kg) (Figure 5D). However, a greater protection was evidently observed in the amygdala, piriform cortex, and entorhinal cortex in animals treated with midazolam (Figure 5D; *P* < .05 vs phenobarbital 60 mg/kg). Overall, these histopathological results suggest that a high‐dose treatment with phenobarbital provides significant protection against DFP‐induced neuronal injury in extrahippocampal regions.

### 3.4 Effect of phenobarbital on DFP‐induced acute degeneration of Nissl‐stained cells in the amygdala and other regions

**Figure 6:**
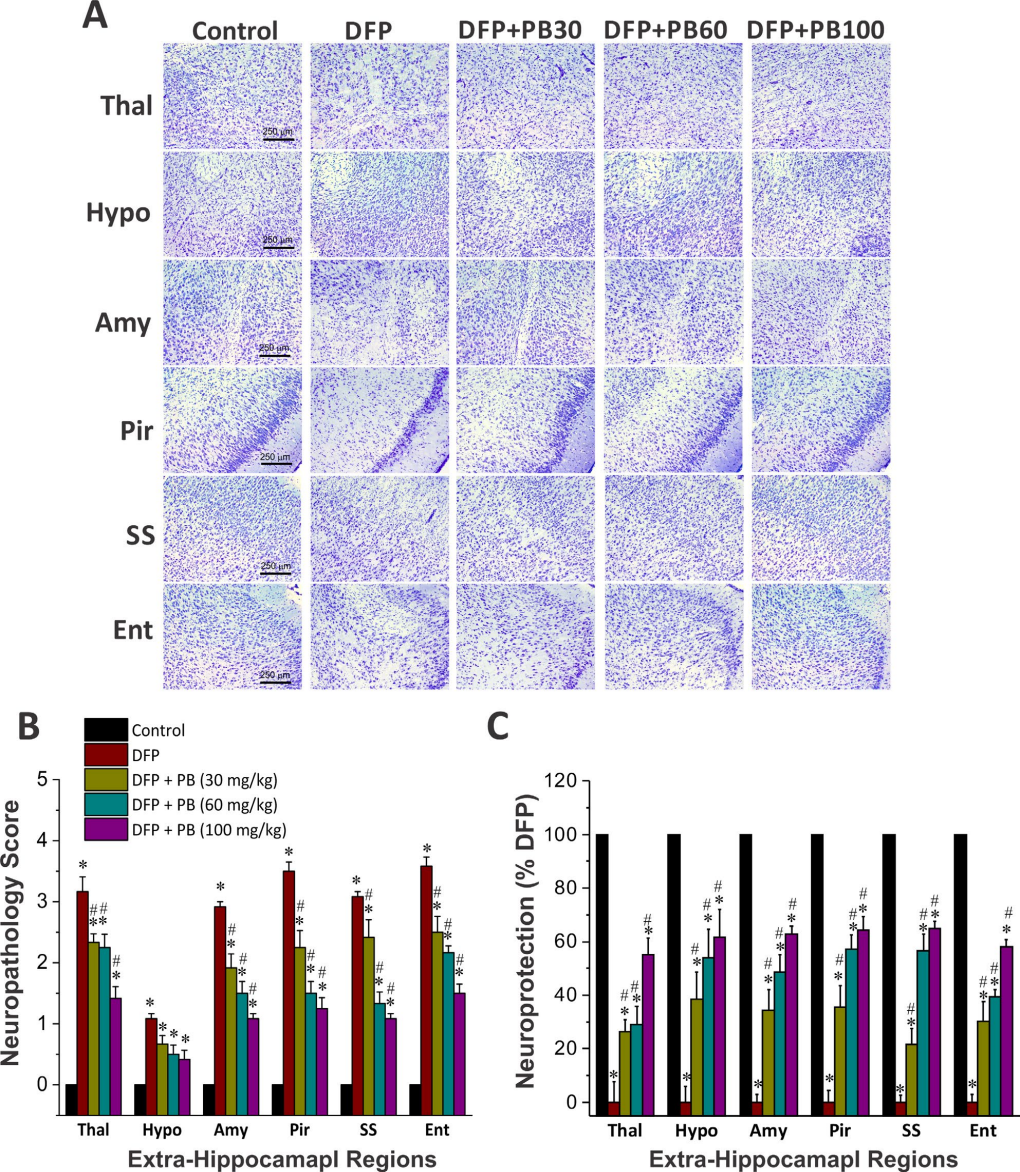
Dose‐dependent protective profile of phenobarbital (PB) on DFP‐induced acute neurodegeneration in the extrahippocampal regions. A, Nissl‐stained images of brain sections from control (n = 8), DFP (n = 6), and phenobarbital (30 mg/kg (n = 5), 60 mg/kg (n = 6), and 100 mg/kg (n = 5)) cohorts. Neurodegeneration is dramatic in many regions in the DFP alone group, which was strikingly reduced in phenobarbital (100 mg/kg) cohort. B, The bar chart depict neuropathology scores in these regions and represent severity level of FJB(+) staining neurons in the absence or presence of phenobarbital at 30, 60 and 100 mg/kg after DFP challenged. DFP exposures were associated with severe damage with high neuropathology score. C, Normalized percent neuroprotection in extrahippocampal brain regions with Nissl staining from rats treated with dosages 40 min post‐DFP. Normalized neuroprotection was calculated using the untreated DFP‐exposed group as the baseline (0% protection). Thal: thalamus; Amy: amygdala; Pir: piriform cortex; Hypo: hypothalamus; Amy: amygdala; SS: somatosensory cortex; Ent: entorhinal cortex. Value in the bar graph represents the mean ± SEM. *P < .05 vs control; #*P* < .05 vs DFP group (Kruskal‐Wallis test followed by the Mann‐Whitney *U* test)

To evaluate whether phenobarbital therapy is associated with protection of principal neurons in the brain, we evaluated brain sections using Nissl staining at 3 days post‐DFP. Nissl staining is a simple histological marker for cells containing Nissl substance, a large extranuclear RNA granular body found in neurons and glia. Nissl staining method is useful to localize the cell body of neuronal cells and non‐neuronal cells. Massive neurodegeneration was evident in the amygdala, thalamus, and cortical regions after DFP exposure (Figure 6). The DPF‐induced neurodegeneration in both hemispheres of the brain appeared mostly symmetrical. There was massive and significant loss of Nissl‐stained neurons (*P* < .05) after DFP exposure in the amygdala, entorhinal, piriform, and somatosensory cortex (Figure 6A,B). There was significant neuroprotection (*P* < .05) at all three doses of phenobarbital as compared to the DFP control group (as evident from minimal cells loss in the phenobarbital groups Figure 6B). However, phenobarbital‐treated subgroup that was treated with the highest dose (100 mg/kg) exhibited the greatest neuroprotection compared to the other two doses (Figure 6B), indicating the neuroprotection of phenobarbital in the DFP model.

## 4 DISCUSSION

We utilized the widely accepted DFP model of OP intoxication in rats to characterize the efficacy and safety of phenobarbital treatment in a delayed (40‐min) postexposure protocol, a paradigm designed to simulate the realistic therapeutic window for drug administration in the scenario of treatment for convulsive status epilepticus and a nerve agent attack on civilians.^16^ Our results demonstrate that phenobarbital protects against seizures and neuropathology in the DFP model. This study found phenobarbital to be a highly effective anticonvulsant against the refractory SE caused by DFP exposure, but was associated with reduced survival rate at higher doses. In addition, phenobarbital facilitated a strong reduction in DFP‐induced neuronal injury in many brain regions. The pattern of negative impact on survival and neurological morbidity are areas of serious concern for phenobarbital therapy. In comparison with diazepam and midazolam, phenobarbital at 60 mg/kg showed comparable survival outcomes.

### 4.1 Lack of effective anticonvulsants for OP intoxication seizures

OP nerve agents are among the most deadly chemical warfare agents that can cause respiratory arrest within minutes of absorption or inhalation. Acute exposure to OPs causes a set of predictable acute toxic signs such as hypersecretion, fasciculations, tremors, convulsions, respiratory distress, and possibly death.^1^, ^2^, ^7^, ^8^ Controlling persistent seizures at an early stage is critical for survival and preventing long‐term neurological dysfunction after OP intoxication. Currently, there are no FDA‐approved postexposure medical countermeasures available to mitigate the effects of OP intoxication.^44^ Available pretreatments (pyridostigmine bromide) and postexposure countermeasures (atropine, 2‐PAM, and diazepam) do not effectively prevent or mitigate all symptoms of nerve agent intoxication.^24^ Furthermore, prophylactic drugs currently in development have limited efficacy and stability and may elicit adverse toxicity after repeated dosing. Thus, there are urgent unmet medical needs to identify effective antidotes or combinations to protect the civilians and soldiers against lethal effects of OP nerve agents. Barbiturates are powerful anticonvulsants and could serve as superior antidotes than benzodiazepines for OP intoxication‐induced seizures and SE. Barbiturates that are positive allosteric modulators of GABA‐A receptors appear to be more effective and long‐lasting than benzodiazepines to control seizures, even when administered very late after OP exposure. Phenobarbital has been shown to mitigate the seizures and lethal effects caused by OP neurotoxicity.^30^, ^31^ However, the overall efficacy and safety of phenobarbital as a delayed (>40 minutes) postexposure anticonvulsant antidote for nerve agents are poorly understood.

### 4.2 Anticonvulsant effects of phenobarbital in OP intoxication seizures

In the present study, phenobarbital rapidly terminated DFP‐induced SE when administered at 40 minutes postexposure. Rats exhibited benzodiazepine resistance at this delayed time point as evident from lack of protection in diazepam‐ and midazolam‐treated groups (Figure 2A,E). Furthermore, phenobarbital‐treated animals exhibited little seizure recurrence after initial suppression, indicating its persistent effectiveness in suppressing the refractory SE. These results reaffirm that phenobarbital can surpass the limitation of benzodiazepines as anticonvulsant antidotes for OP intoxication.^23^, ^24^, ^45^ Here, we utilized DFP as a surrogate chemical agent because it replicates many features of soman or sarin neurotoxicity due to its chemical and mechanistic similarities.^6^, ^18^, ^33^, ^34^ The seizures and SE caused by DFP are highly resistant to the benzodiazepines, diazepam and midazolam (Figure 2A,E), as confirmed in our earlier studies.^24^, ^25^ The average latency of 40‐50 minutes for complete termination of SE following phenobarbital administration is consistent with results from new anticonvulsants such as neurosteroids and glutamate receptor antagonists in nerve agent models.^15^, ^23^ Such robust anticonvulsant efficacy of phenobarbital is not surprising given its powerful allosteric and direct activation actions at GABA‐A receptors in the brain.^46^ Taken together, these observations reinforce the clinical potential of phenobarbital as an alternative postexposure anticonvulsant for nerve agent exposure.

### 4.3 Neuroprotective effects of phenobarbital in OP intoxication neurotoxicity

Neuronal injury and neurodegeneration are hallmark features of OP intoxication.^47^ Neuropathology investigations from DFP and soman models revealed extensive loss of principal neurons and interneurons in the hippocampus, amygdala, and cortical regions.^6^, ^40^, ^47^, ^48^, ^49^, ^50^, ^51^ The temporal‐spatial patterns of neuronal injury have strong impact on drug therapies for controlling SE caused by OP intoxication. OP exposure downregulates critical inhibitory GABA‐A receptors, kills neurons, and causes massive neuroinflammation that will cause more neuronal death, which worsens the problem of too few benzodiazepine receptors.^25^ The loss of inhibitory interneurons creates a self‐sustaining seizure circuit and refractory SE. Thus, a medical countermeasure with neuroprotection property is desirable for effective management of OP intoxication. In the present study, phenobarbital treatment is associated with a dose‐related neuroprotection against DFP‐induced acute neuronal injury and necrosis as revealed by FJB histology and stereology quantification of injured neuronal counts. This neuroprotective effect is far superior to diazepam and midazolam at 40‐60 minutes post‐DFP treatment windows (Figures 2E and 4D‐F).^24^, ^25^ Therefore, it is highly likely that such protection may underline phenobarbital's ability to rescue extensive damage of principal cells and interneurons from the OP neurotoxicity, especially to limit or prevent the long‐term neurological dysfunction or risk for the development of chronic epilepsy after refractory SE. Regarding the potential influence of selection bias of surviving rats on neuroprotection outcomes, it is unlikely to be a major caveat for the neuroprotection study done at 3 days after DFP exposure. The greater mortality with phenobarbital is mainly attributed to cardiovascular comprise, not due to seizures or related neurotoxicity. Animals treated with the benzodiazepines midazolam and diazepam, despite showing comparable survival rate to that of phenobarbital at 60 mg/kg, did not exhibit comparable neuroprotection outcomes. Therefore, the selection bias of surviving cohort is not a caveat for the neuroprotection assessment. Additionally, benzodiazepines such as diazepam and midazolam can be used for inpatient and prehospital settings with better clinical benefits.^16^ This is consistent with our previously reported findings which show that early benzodiazepine administration at 10 minutes post‐DFP is associated with better outcomes than delayed therapy at 40 minutes post‐DFP.^24^, ^25^ Overall, our results suggested that early administration of benzodiazepines at or before 10 minutes and late administration of phenobarbital at 40 minutes would produce beneficial effects in attenuating functional seizure activity as well as structural neuroprotection during SE management.^16^, ^24^, ^25^

### 4.4 The overall risk‐benefit ratio of phenobarbital therapy in OP intoxication

Barbiturates are the drugs of choice for treatment of refractory SE because they are easy to use, produce rapid onset, elicit a strong and persistent action due to their long half‐life, and are available in convenient formulations.^26^ Phenobarbital is used as second‐line drug for refractory SE, irrespective of the seizure etiology. Phenobarbital has been tested in a few animal models of OP intoxication.^30^, ^31^, ^32^ Based on overall survival rate, functional seizure suppression, and histological neurodegeneration outcomes, phenobarbital at 60 mg/kg may be a better option of the other two phenobarbital doses in DFP‐induced epilepsy, especially for ambulatory treatment. Certain limitations exist with phenobarbital therapy such as strong sedation, pharmacokinetic interactions, potential impairment of renal and cardiac function, and longer action leading to neurological morbidity or comatose condition, especially at higher concentration such as 100 mg/kg in our experiment. Furthermore, there is paucity of information on overall risk‐benefit ratio of phenobarbital therapy in controlling the OP‐induced refractory SE.

The steep dose‐adverse effect relationship associated with phenobarbital in SE therapy is linked to its mode of action at GABA‐A receptors and other targets in the brain.^52^ The progression of neurological adverse outcomes of phenobarbital is dose‐dependent and evidently much serious at the higher doses that are effective to terminate SE. Patients with seizures treated with phenobarbital at serum concentrations of >300 mg/L have benefited from further dose increases, with preservation of respiratory drive, adequate minute ventilation, and close monitoring of phenobarbital therapeutic range.^53^ There are certain caveats to this interpretation or any type of adverse safety data from animal models that is intended to inform human risk assessment. The context of the investigated dose(s) relative to pharmacokinetics and to potential human therapeutic dose of concern should be discerned further toward formulation risk mitigation, especially in the hospital settings. Nevertheless, a careful risk assessment and monitoring program, such as the FDA's approval of a Risk Evaluation and Mitigation Strategy, may limit the concerns about serious risks, including excessive sedation or cardio‐respiratory impairment during phenobarbital administration. This mitigation program is only available to patients through a restricted distribution program at certified healthcare facilities where the healthcare provider can carefully monitor the patient. The anesthetic ketamine, which is being proposed as anticonvulsant for refractory SE, may have similar limitations as phenobarbital.^54^ In addition, the potential biological variability including sex differences in the protective effect of phenobarbital warrant further scrutiny.^55^ Moreover, a combination study of phenobarbital and midazolam would provide key insights on the potential synergistic or additive protective outcomes. Such experiments will be considered in the future. Recently, we completed an isobolographic study on the combination potential of the GABA‐A receptor‐modulating neurosteroids (brexanolone and ganaxolone) with midazolam in SE models.^56^ Our results show a synergistic protective effect of neurosteroid‐midazolam combination.^56^

In conclusion, these results are consistent with recent reports in nerve agent models^31^, ^32^ and confirm that phenobarbital is an effective anticonvulsant for controlling DFP‐induced, benzodiazepine‐refractory SE when given at or after 40 minutes. It has significant protective effect against DFP‐induced massive neuronal injury and degeneration, indicating its neuroprotectant action. However, phenobarbital therapy is associated with negative overall outcomes including serious cardio‐respiratory dysfunction and anesthetic or comatose state that are strong indicators of its potential to cause serious neurological adverse effects at the doses needed to suppress refractory SE. A careful risk‐benefit analysis is essential to consider phenobarbital as an alternative anticonvulsant for OP intoxication, especially for its use in mass casualty situations.

## CONFLICT OF INTEREST

The authors have no competing financial interests. We confirm that we have read the journal's position on issues involved in ethical publication and affirm that this report is consistent with those guidelines.
